# Subclinical hypothyroidism increases insulin resistance in normoglycemic people

**DOI:** 10.3389/fendo.2023.1106968

**Published:** 2023-07-06

**Authors:** Wanyu Yang, Chenye Jin, Haoyu Wang, Yaxin Lai, Jiashu Li, Zhongyan Shan

**Affiliations:** ^1^ Department of Geriatric Medicine, The First Hospital of China Medical University, Shenyang, Liaoning, China; ^2^ Department of Rheumatology Immunology, The First Hospital of China Medical University, Shenyang, Liaoning, China; ^3^ Department of Endocrinology and Metabolism and The Institute of Endocrinology, National Health Commission (NHC) Key Laboratory of Diagnosis and Treatment of Thyroid Diseases, The First Hospital of China Medical University, Shenyang, Liaoning, China

**Keywords:** thyroid stimulating hormone, subclinical hypothyroidism, thyroid hormone sensitivity, diabetes, insulin resistance

## Abstract

**Objective:**

To investigate the effect of simple subclinical hypothyroidism (SCH) and type 2 diabetes mellitus (T2DM) combined with SCH on insulin resistance.

**Design and methods:**

A total of 622 people with newly diagnosed T2DM were selected as the study subjects, and 621 normoglycemic people were selected as control subjects. According to the diagnostic criteria of thyroid diseases, the subjects were divided into a normal thyroid function group and a subclinical hypothyroidism group. Both groups received a physical examination, and blood samples were collected. The measurement indexes included FPG, FINS, OGTT2hPG, OGTT2hINS, HbA1c, TC, TG, HDL-C, LDL-C, TSH, FT3 and FT4. HOMA-IR, HOMA-β, and TFQI (thyroid feedback quantile index) were calculated.

**Results:**

There was no significant difference in age or sex distribution between the T2DM group and the normoglycemic group (P>0.05). The prevalence of thyroid dysfunction in the T2DM group was significantly higher than that in the normoglycemic group (16.39% vs. 11.27%, P<0.05), and among the different types of thyroid dysfunction, the prevalence of SCH was the highest at 14.95% (P<0.05). There was no significant difference in BMI, waist-hip ratio, blood lipid profile, HOMA-β, and HOMA-IR values between the T2DM with subclinical hypothyroidism group (T2DM+SCH+ group) and the normal thyroid function group (T2DM+SCH- group) (P>0.05). The BMI, waist-hip ratio and HOMA-IR values of the normoglycemic group with subclinical hypothyroidism (T2DM-SCH+ group) were significantly higher than those of the normoglycemic group with normal thyroid function (T2DM-SCH- group) (P<0.05), and there were no significant differences between the T2DM+SCH- and T2DM+SCH+ groups (P>0.05). HOMA-β values were significantly higher in the T2DM-SCH+ group than in the T2DM-SCH-, T2DM+SCH- and T2DM+SCH+ groups (P<0.05). As the TFQI value increased, the body weight, waist-hip ratio, diastolic blood pressure, FPG, OGTT2hPG and HbA1c values gradually increased in the T2DM group and normoglycemic group (P<0.05). HDL-C, FINS, OGTT2hINS and HOMA-β values gradually decreased (P<0.05).

**Conclusion:**

Subclinical hypothyroidism only increases insulin resistance in normoglycemic people. As the sensitivity of the central thyroid decreases, the risk of developing diabetes increases.

## 1 Introduction

At present, the incidence of diabetes mellitus (DM) is rapidly increasing worldwide. Diabetes has become an important public health problem worldwide. According to the latest epidemiological survey in China, the prevalence of diabetes is 12.8%, and the number of people aged 18 years and above with diabetes has reached 129.8 million (70.4 million males and 59.4 million females) (1). Type 2 diabetes mellitus (T2DM) is a complex condition that results from interactions between genetic factors and the environment. Insulin resistance and insulin secretion decline are the core features of T2DM.

Diabetes and thyroid disease are two of the most common types of endocrine metabolic diseases. A large amount of data shows a strong relationship between diabetes and thyroid disease. The proportion of patients with T2DM with thyroid disease is significantly higher than that of healthy people (2–4), and subclinical hypothyroidism (SCH) accounts for the largest proportion of these cases (5, 6). SCH is a pathological condition in which the levels of thyroid stimulating hormone (TSH) in the blood are increased and the levels of thyroid hormones (FT3 and FT4) are in the normal range. The thyroid feedback quantile index (TFQI) is a newly calculated thyroid hormone central sensitivity index and quantifies the deviation of the pituitary response to thyroid hormone in a continuous manner (7). The TFQI converts the probability distributions of TSH and FT4 into a probability quantile between 0 and 1 through the principle of the empirical cumulative distribution function. After calculating the formula, the TFQI values range from -1 to 1. The advantage of this index is that it does not produce extreme values in the case of thyroid dysfunction and is relatively stable.

SCH can affect lipid metabolism, insulin sensitivity and other aspects and cause a series of metabolic disorders. A study by Eirini Maratou found that SCH patients had an increased insulin resistance index and a decreased insulin sensitivity index compared with those of individuals with normal thyroid function (8). Another case-control study similarly suggested the same results.Compared to the control group.Homeostatic model assessment of insulin resistance (HOM-IR) significantly elevated in patients with SCH (9).However, other studies have found that SCH is not associated with the insulin resistance index (10). Therefore, the correlation between SCH and insulin resistance needs to be further explored. Clarifying the relationship between SCH and insulin resistance will lead to a better understanding of the pathogenesis and treatment of thyroid disease and diabetes, therefor we designed the current clear and simple clinical study with sufficient sample size to investigate the mentioned correlations properly.The aim of this study was to investigate the effects of SCH alone and T2DM combined with SCH on insulin resistance.

## 2 Materials and methods

### 2.1 Study population

The case group included 622 patients who were newly diagnosed with T2DM according to 1999 WHO criteria and attended the outpatient clinic of the Department of Endocrinology and Metabolic Diseases, the First Affiliated Hospital of China Medical University between December 2018 to December 2019. These patients had a course of disease of less than 12 months, did not use hypoglycemic drugs, or had a history of medication of less than 1 month, and they did not use drugs in the 3 months before enrollment. These patients also had a glycosylated hemoglobin (HbA1c) level between 7% and 10% and a fasting plasma glucose (FPG) level < 11.1 mmol/L. There were 621 people in the normoglycemic group from the community epidemiological survey population in Shenyang, Liaoning, China, during the same time period. For both groups, the exclusion criteria were as follows: severe heart, liver, and kidney diseases and obvious hematological diseases; pregnancy, expecting to become pregnant or breastfeeding; previous thyroid disease and use of drugs that affect thyroid function (including thyroid hormone supplementation and antithyroid medications); and the use of insulin for the treatment of diabetic ketoacidosis and hyperosmolar nonketotic coma. This study was approved by the ethics committee, and all subjects signed informed consent forms.

### 2.2 Data collection

Detailed information on medical history, medication history and other general conditions was collected. Both groups underwent physical examination and blood sampling. The physical examination included height (cm), weight (kg), waist circumference (cm), hip circumference (cm), and blood pressure (mm/hg) measurements. Fasting venous blood was collected, and fasting plasma glucose (FPG, 3.90-6.10 mmol/L), fasting insulin (FINS, 4.03-23.46 mIU/L), glycosylated hemoglobin (HbA1c, 3.90%-6.10%), total cholesterol (TC, 0.00-5.72 mmol/L), triglyceride (TG, 0.00-1.70 mmol/L), high-density lipoprotein cholesterol (HDL-C, 0.91-1.92 mmol/L), low-density lipoprotein cholesterol (LDL-C, 0.00-3.64 mmol/L), thyroid-stimulating hormone (TSH), free triiodothyronine (FT3), and free thyroxine (FT4) levels were measured. All participants underwent a 75 g oral glucose tolerance test to measure 2-hour intravenous plasma glucose (OGTT2hPG) and insulin (OGTT2hINS) levels. Homeostasis model assessment of insulin resistance (HOMA-IR), islet β function index (HOMA-β), thyroid feedback quantile index (TFQI, values range from -1 to 1), body mass index (BMI) and waist-hip ratio (WHR) were calculated. The relevant indexes were calculated as follows: BMI = weight/height^2^; WHR = waist/hip circumference; HOMA-IR = fasting plasma glucose*fasting insulin/22.5; HOMA-β = 20*fasting insulin/(fasting plasma glucose-3.5); TFQI = cdfFT4 - (1- cdfTSH).

### 2.3 Laboratory measurements

TSH, FT3, and FT4 levels were measured by an electrochemiluminescence immunoassay (Cobas Elesys601, Roche Diagnostics, Switzerland). Plasma glucose levels were measured by the hexokinase endpoint method (Olympus, Japan), and glycosylated hemoglobin levels were measured by Bio-Rad reagent (Bio-Rad, USA). Serum insulin levels were determined by the chemiluminescence method using a Maglumi2000 automatic luminescence immunoanalyzer (Shenzhen New Industry Kit, China), and serum lipid levels (TC, TG, HDL-C, and LDL-C) were determined by Mindray (BS-180) reagent (Mindray, China).

### 2.4 Diagnostic criteria

Subclinical hypothyroidism: TSH>4.2 mIU/L, FT4 normal (12-22 pmol/L). Subclinical hyperthyroidism: TSH<0.27 mIU/L, FT4 normal, FT3 normal (3.1-6.8 pmol/L). Clinical hypothyroidism: TSH>4.2 mIU/L, FT4<12 pmol/L. Clinical hyperthyroidism: TSH<0.27 mIU/L, FT4>22 pmol/L and/or FT3>6.8 pmol/L.

### 2.5 Study population grouping

According to the diagnostic criteria of T2DM and thyroid diseases, the subjects were divided into a normoglycemic thyroid function group (T2DM-SCH- group), normoglycemic subclinical hypothyroidism group (T2DM-SCH+ group), T2DM euthyroidism group (T2DM+SCH- group), and subclinical hypothyroidism group (T2DM+SCH+ group) for people with T2DM.

### 2.6 Statistical analyses

**Figure 1:**
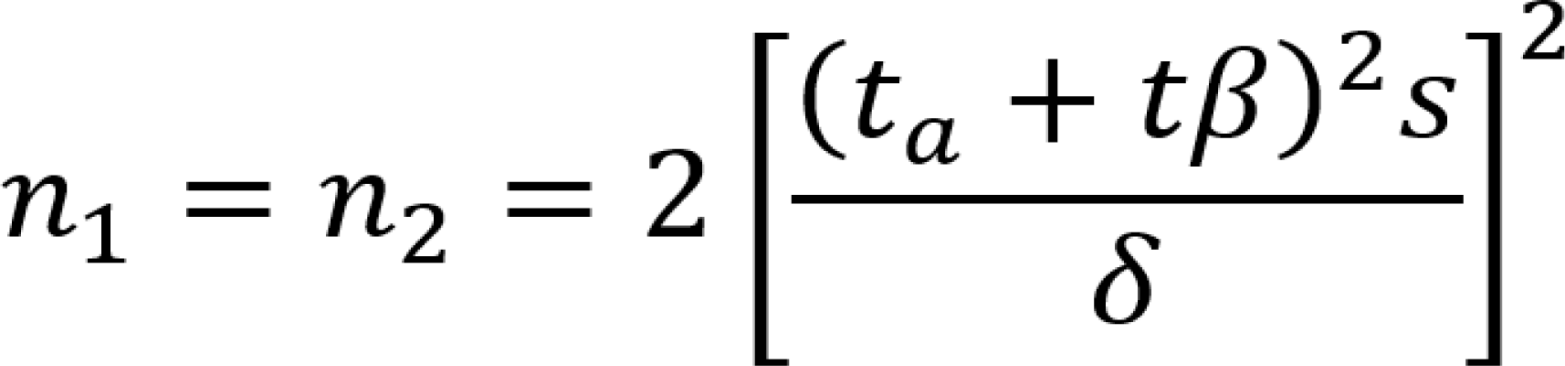
Sample size calculation formula.

The PASS software was used to calculate the statistical power and determine the sample size (11) (**Figure 1**).All data were entered into an Excel spreadsheet and used for statistical analysis using SPSS 19.0 software. The measurement data conforming to a normal distribution are expressed as the “mean ± standard deviation”. The independent sample T test was used for comparisons between two groups, and ANOVA was used for comparisons among more than two groups. The nonnormally distributed measurements are expressed as medians (interquartile ranges), and the Mann−Whitney test was used for comparisons between groups. The count data are expressed as quantities and percentages. The comparison of count data rates and composition ratios was performed by chi-square analysis, and correlation and multiple linear regression analysis were applied for correlation analysis. P<0.05 indicated that a difference was statistically significant.

## 3 Results

### 3.1 Comparison of general data between the normoglycemic group and the type 2 diabetes mellitus group

The demographic, anthropometric and biochemical parameters were compared between the normoglycemic (N=621) and T2DM individuals (N=622). As shown in **Table 1**, there were no significant differences in age, sex composition or blood pressure between the two groups (P>0.05). BMI, WC, HC, WHR, TC, TG, FPG, OGTT2hPG, HbA1c, HOMA-IR and TFQI values in the T2DM group were significantly higher than those in the normoglycemic group (P<0.05). HDL-C, FINS, OGTT2hINS and HOMA-β values in the T2DM group were significantly lower than those in the normoglycemic group (P<0.05).

**Table 1:** Comparison of general data between the normoglycemic group and T2DM group.

	normoglycemic	T2DM	p
Participants	621	622	
Age	49.61 ± 6.79	50.41 ± 8.79	0.07
Male/Female	307/314	328/294	0.25
BMI (kg/m^2^)	24.69 ± 3.26	25.68 ± 2.59*	**<0.05**
WC (cm)	85.57 ± 9.89	89.07 ± 8.39*	**<0.05**
HC (cm)	96.98 ± 6.57	98.77 ± 7.52*	**<0.05**
WHR	0.88 ± 0.07	0.90 ± 0.05*	**<0.05**
SBP (mm/Hg)	124.34 ± 17.40	124.59 ± 12.38	0.77
DBP (mm/Hg)	80.49 ± 10.69	80.20 ± 7.78	0.58
TC (mmol/L)	5.06 ± 1.02	5.25 ± 1.12*	**<0.05**
TG (mmol/L)	1.88 ± 1.63	2.49 ± 2.58*	**<0.05**
HDL-C (mmol/L)	1.39 ± 0.36	1.23 ± 0.30*	**<0.05**
LDL-C (mmol/L)	3.12 ± 0.86	3.04 ± 0.90	0.12
FPG (mmol/L)	5.30 (5.00,5.60)	8.07 (7.27,9.41)*	**<0.05**
OGTT2hPG (mmol/L)	6.60 (5.70,7.80)	12.45 (10.40,14.70)*	**<0.05**
HbA1c (%)	5.70 ± 0.45	8.25 ± 0.93*	**<0.05**
FINS (pmol/L)	15.11 (11.48,20.14)	10.92 (7.34,16.30)*	**<0.05**
OGTT2hINS (pmol/L)	60.11 (38.02,92.02)	31.39 (20.74,44.23)*	**<0.05**
HOMA-β	172.92 (125.72,229.69)	48.43 (28.45,72.87)*	**<0.05**
HOMA-IR	3.59 (2.62,4.85)	3.94 (2.56,6.12)*	**<0.05**
TSH (mIU/L)	1.97 (1.35,2.90)	2.20 (1.43,3.31)*	**<0.05**
FT3 (pmol/L)	4.89 ± 0.97	5.30 ± 0.64*	**<0.05**
FT4 (pmol/L)	16.07 ± 2.60	17.78 ± 2.44*	**<0.05**
TFQI	-0.16 (-0.30,0.00)	0.08 (-0.12,0.26)*	**<0.05**

BMI, body mass index; WC, waist circumference; HC, hip circumference; WHR, waist-hip ratio; SBP/DBP, systolic/diastolic blood pressure; TC, total cholesterol; TG, triglycerides; HDL-C/LDL-C, high-density/low-density lipoprotein cholesterol; FPG, fasting blood glucose; OGTT2hPG, 2h intravenous plasma glucose; HbA1c, glycosylated hemoglobin; FINS, fasting insulin; OGTT2hINS, 2h intravenous plasma insulin; HOMA-β, Homeostasis model assessment of islet β function index; HOMA-IR, Homeostasis model assessment of insulin resistance;TSH, thyroid-stimulating hormone; FT3, free triiodothyronine; FT4, free thyroxine;TFQI, thyroid feedback quantile index.*Compared to the normoglycemic P<0.05. Bold values: Compared to the normoglycemic P<0.05.

### 3.2 Prevalence of thyroid disease in the normoglycemic and T2DM groups

The levels of thyroid function indicators and thyroid dysfunction prevalence were compared between the groups with or without T2DM. As shown in **Table 2**, the prevalence of thyroid dysfunction in the T2DM group was significantly higher than that in the normoglycemic group (16.39% vs. 11.27%, P<0.05), and among the different types of thyroid dysfunction, the prevalence of SCH was the highest at 14.95% (P<0.05).

**Table 2:** Prevalence of thyroid disease in the normoglycemic and T2DM group.

	Normoglycemic N (% )	T2DM N (% )	p
Participants	621	622	
Thyroid dysfunction	70 (11.27%)	102 (16.39%)*	**<0.05**
Subclinical hypothyroidism	54 (8.70%)	93 (14.95%)*	**<0.05**
Clinical hypothyroidism	10 (1.61%)	5 (0.80%)	0.193
Subclinical hyperthyroidism	1 (0.16%)	2 (0.32%)	0.564
Clinical hyperthyroidism	5 (0.80%)	2 (0.32%)	0.255

*Compared to the normoglycemic P<0.05. Bold values: Compared to the normoglycemic P<0.05.

### 3.3 Comparison of general, anthropometric and biochemical characteristics in subjects with different TFQI values

As the TFQI value increased, the BMI, WC, HC, WHR, DBP, FPG, OGTT2hPG and HbA1c values of the two groups gradually increased (P<0.05). HDL-C, FINS, OGTT2hINS and HOMA-β values gradually decreased (P<0.05) (**Table 3**).

**Table 3:** Comparison of data in subjects with different TFQI values.

	TFQI			
	Q1 (-1≤TFQI≤-0.35)	Q2 (-0.35<TFQI ≤ 0.43)	Q3 (0.43<TFQI ≤ 1)	P
Age	48.92 ± 6.40	50.13 ± 8.01	50.67 ± 8.33	0.19
BMI (kg/m2)	24.95 ± 3.19	25.19 ± 2.97	26.07 ± 2.38	0.11
WC (cm)	85.79 ± 10.20	87.37 ± 9.21	91.74 ± 7.82	**<0.05**
HC (cm)	97.56 ± 7.33	97.78 ± 7.00	101.73 ± 8.56	**<0.05**
WHR	0.88 ± 0.07	0.89 ± 0.06	0.90 ± 0.05	**<0.05**
SBP (mm/Hg)	121.80 ± 15.34	124.83 ± 15.12	124.65 ± 12.67	0.07
DBP (mm/Hg)	78.40 ± 9.42	80.63 ± 9.37	80.03 ± 7.47	**<0.05**
TC (mmol/L)	5.11 ± 1.06	5.17 ± 1.07	4.89 ± 1.14	0.21
TG (mmol/L)	2.02 ± 2.09	2.20 ± 2.22	2.41 ± 1.44	0.51
HDL-C (mmol/L)	1.37 ± 0.36	1.31 ± 0.34	1.16 ± 0.32	**<0.05**
LDL-C (mmol/L)	3.07 ± 0.89	3.09 ± 0.88	2.83 ± 1.01	0.17
FPG (mmol/L)	5.40 (5.10,6.30)	6.40 (5.30,8.14)	7.80 (7.10,9.24)	**<0.05**
OGTT2hPG (mmol/L)	7.30 (5.90,9.70)	9.10 (6.60,12.70)	12.10 (10.20,14.20)	**<0.05**
HbA1c (%)	6.35 ± 1.21	7.02 ± 1.47	8.07 ± 1.11	**<0.05**
FINS (pmol/L)	14.14 (9.98,20.40)	13.30 (8.80,18.26)	9.04 (6.50,14.74)	**<0.05**
OGTT2hINS (pmol/L)	50.71 (32.58,81.58)	41.41 (25.60,65.36)	27.08 (22.06,33.95)	**<0.05**
HOMA-β	157.47 (86.48,214.00)	90.73 (46.26,171.86)	41.55 (29.16,63.89)	**<0.05**
HOMA-IR	3.62 (2.60,5.25)	3.74 (2.62,5.39)	3.15 (2.43,5.16)	0.37

BMI, body mass index; WC, waist circumference; HC, hip circumference; WHR, waist-hip ratio;SBP/DBP, systolic/diastolic blood pressure;TC, total cholesterol; TG, triglycerides; HDL-C/LDL-C, high-density/low-density lipoprotein cholesterol; FPG, fasting blood glucose; OGTT2hPG, 2h intravenous plasma glucose; HbA1c, glycosylated hemoglobin; FINS, fasting insulin; OGTT2hINS, 2h intravenous plasma insulin; HOMA-β, Homeostasis model assessment of islet β function index; HOMA-IR, Homeostasis model assessment of insulin resistance. Bold values: There were differences in TFQI among the three groups.

### 3.4 Comparison of insulin resistance in subjects with or without SCH

There were no significant differences in BMI, WC, HC, WHR, TC, TG, LDL-C, HDL-C, FINS, OGTT2hINS, HOMA-β, or HOMA-IR values between the T2DM+SCH+ group and the T2DM+SCH- group (P>0.05) (**Table 4**), suggesting that SCH did not increase insulin resistance in newly diagnosed T2DM patients.

**Table 4:** Comparison of insulin resistance in subjects with or without SCH.

	normoglycemic		T2DM	
	Normal thyroid function	SCH	Normal thyroid function	SCH
Participants	551	54	520	93
Age	49.54 ± 6.71	50.25 ± 7.54	49.84 ± 8.64	53.60 ± 9.02
BMI (kg/m2)	24.60 ± 3.21	25.70 ± 3.61^a^	25.75 ± 2.61^a^	25.29 ± 2.47^a^
WC (cm)	85.30 ± 9.78	88.40 ± 10.64^a^	89.13 ± 8.57^a^	88.72 ± 7.32^a^
HC (cm)	96.86 ± 6.58	98.26 ± 6.46	98.82 ± 7.66^a^	98.52 ± 6.72^a^
WHR	0.88 ± 0.07	0.90 ± 0.07^a^	0.90 ± 0.05^a^	0.90 ± 0.05^a^
SBP (mm/Hg)	124.16 ± 17.61	126.23 ± 15.06	124.30 ± 12.28	126.26 ± 12.90
DBP (mm/Hg)	80.39 ± 10.81	81.61 ± 9.33	80.27 ± 7.72	79.80 ± 8.14
TC (mmol/L)	5.08 ± 1.00	4.90 ± 1.14	5.25 ± 1.06^ab^	5.24 ± 1.41
TG (mmol/L)	1.88 ± 1.63	2.00 ± 1.61	2.43 ± 2.29^a^	2.82 ± 3.83^ab^
HDL-C (mmol/L)	1.40 ± 0.36	1.33 ± 0.33	1.23 ± 0.29^ab^	1.25 ± 0.35^a^
LDL-C (mmol/L)	3.13 ± 0.86	3.00 ± 0.88	3.08 ± 0.88	2.89 ± 1.03^a^
FPG (mmol/L)	5.30 (5.00,5.60)	5.40 (5.08,5.73)	8.10 (7.27,9.46)^ab^	7.91 (7.35,9.02)^ab^
OGTT2hPG (mmol/L)	6.50 (5.60,7.70)	7.05 (6.00,8.08)	12.40 (10.41,14.71)^ab^	12.75 (10.35,14.67)^ab^
HbA1c (%)	5.70 ± 0.45	5.77 ± 0.43	8.25 ± 0.93^ab^	8.26 ± 0.93^ab^
FINS (pmol/L)	14.99 (11.26,19.70)	17.37 (13.29,24.29)^a^	10.87 (7.43,16.25)^ab^	11.17 (6.67,16.45)^ab^
OGTT2hINS (pmol/L)	57.90 (35.94,88.10)	80.74 (56.40,105.44)^a^	31.26 (20.82,44.10)^ab^	32.32 (19.29,45.47)^ab^
HOMA-β	171.86 (124.08,228.44)	188.17 (147.14,263.55)^a^	48.62 (27.99,72.48)^ab^	46.88 (30.55,74.35)^ab^
HOMA-IR	3.55 (2.61,4.74)	4.15 (3.32,5.75)^a^	3.93 (2.55,6.05)^a^	3.96 (2.55,6.28)^a^
TSH (mIU/L)	1.84 (1.27,2.58)	5.67 (4.83,7.60)^a^	1.99 (1.34,2.72)^bc^	5.36 (4.65,6.64)^a^
FT3 (pmol/L)	4.91 ± 0.99	4.76 ± 0.57	5.32 ± 0.65^abc^	5.12 ± 0.60^ab^
FT4 (pmol/L)	16.18 ± 2.65	14.96 ± 1.68^a^	17.90 ± 2.61^abc^	16.36 ± 2.25^b^
TFQI	-0.18 (-0.32,-0.01)	-0.02 (-0.16,0.10)^a^	0.08 (-0.13,0.25)^abc^	0.08 (-0.10,0.34)^ab^

BMI, body mass index; WC,waist circumference; HC,hip circumference; WHR,waist-hip ratio; SBP/DBP, systolic/diastolic blood pressure; TC, total cholesterol; TG, triglycerides; HDL-C/LDL-C, high-density/low-density lipoprotein cholesterol; FPG, fasting blood glucose; OGTT2hPG, 2h intravenous plasma glucose; HbA1c, glycosylated hemoglobin; FINS, fasting insulin; OGTT2hINS, 2h intravenous plasma insulin; HOMA-β , Homeostasis model assessment of islet β function index; HOMA-IR, Homeostasis model assessment of insulin resistance.TSH, thyroid-stimulating hormone; FT3,free triiodothyronine; FT4, free thyroxine; TFQI, thyroid feedback quantile index; ^a^Compared with T2DM-SCH-group, P< 0.05. ^b^Compared with T2DM-SCH+group,P< 0.05. ^c^Compared with T2DM+SCH+ group,P< 0.05.

The BMI, WC, WHR and HOMA-IR values in the T2DM-SCH+ group were significantly higher than those in the T2DM-SCH- group (P<0.05), but no significant difference was found between the T2DM+SCH- group and the T2DM+SCH+ group (P>0.05). These findings suggest that T2DM-SCH+ group have insulin resistance similar to those in T2DM.

The FINS, OGTT2hINS and HOMA-β values in the T2DM-SCH+ group were significantly higher than those in the T2DM-SCH-, T2DM+SCH- and T2DM+SCH+ groups (P<0.05). These findings suggest that subclinical hypothyroidism insulin compensates for the increase in normal blood glucose levels.

### 3.5 Multiple regression analysis of HOMA-IR in euthyroid and SCH groups within normoglycemic subjects

**Figure 2:**
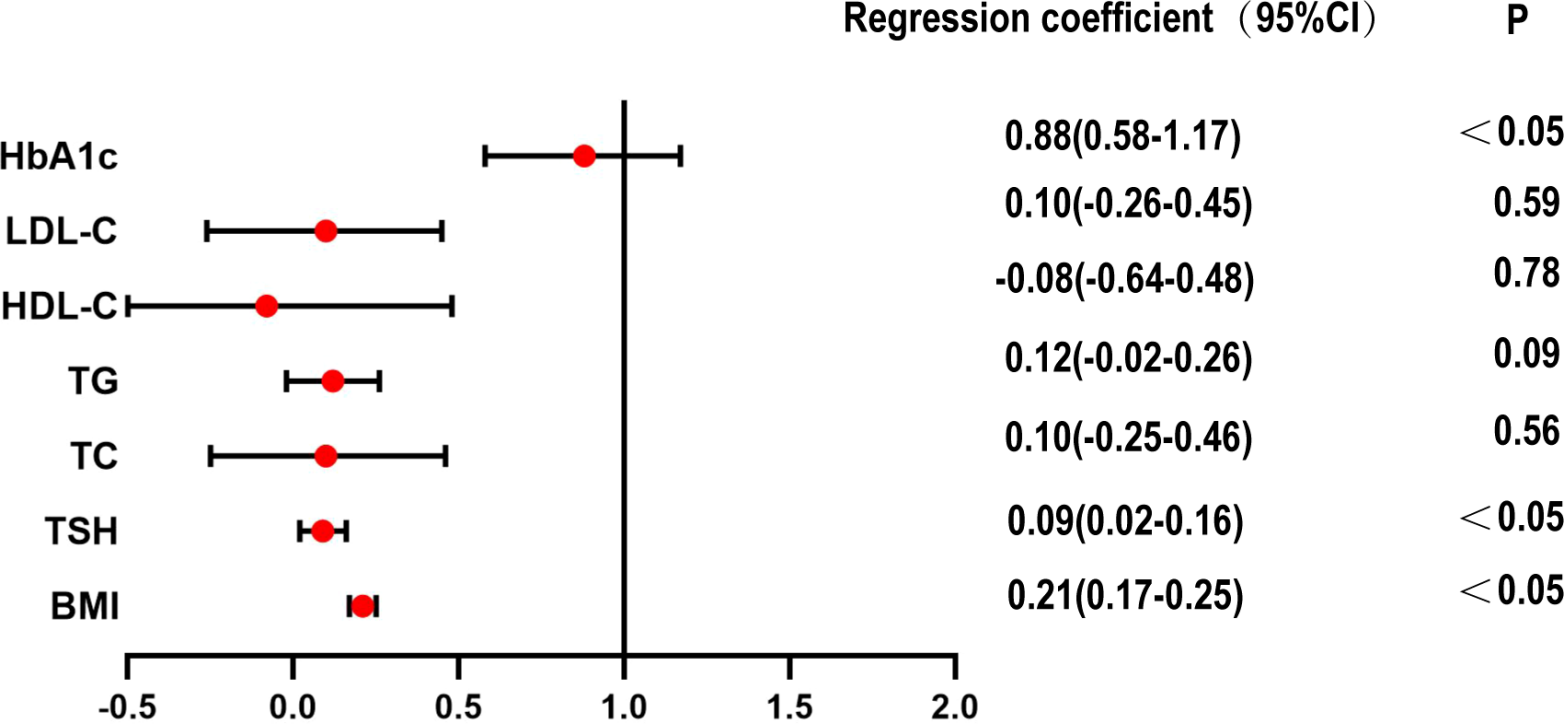
Multiple regression analysis of HOMA-IR in euthyroid and SCH group within normoglycemic subjects.

In the case of normal blood glucose levels, multiple regression analysis of HOMA-IR in the euthyroid and SCH groups showed that BMI, TSH and HbA1c were risk factors for increased HOMA-IR (**Figure 2**).

Correlation analysis of the euthyroid subgroup and subclinical hypothyroidism subgroup in the T2DM group showed that there was no obvious correlation between TSH and HOMA-IR.

## 4 Discussion

This cross-sectional study of a Chinese population with normoglycemia and T2DM found that the prevalence of thyroid dysfunction in the T2DM group was significantly higher than that in the control group, and the prevalence of SCH was the highest among different types of thyroid dysfunction. SCH only increased insulin resistance in the normoglycemic population but did not increase insulin resistance in the population with T2DM. In this population, a decrease in central thyroid sensitivity increases the risk of developing diabetes.

T2DM is a metabolic disorder syndrome characterized by insulin resistance and/or insufficient secretion of islet β cells, and there are many factors involved in its occurrence and development. Studies have found (12, 13) that the occurrence of diabetes is also accompanied by the occurrence of other diseases, such as hypertension, obesity, thyroid disease and sleep apnea syndrome, and diabetes combined with thyroid disease has attracted increasing attention from many scholars in recent years. Numerous studies suggest a significantly increased prevalence of thyroid disease in people with diabetes compared with healthy people (2, 3). The results of this paper suggest that the prevalence of thyroid disease is significantly higher in the population with diabetes than in the population without diabetes (16.39% vs. 11.27%). Among this population, subclinical hypothyroidism was the most common form of thyroid disease, with a prevalence of 14.95%. This finding is consistent with that from previous studies (5, 6, 14). Leptin is an important neuroendocrine regulator of the hypothalamic-pituitary-thyroid (HPT) axis. It not only directly regulates the expression of the paraventricular nucleotropin-releasing hormone (TRH) gene but also indirectly regulates TRH by influencing the arcuate nucleus (ARC) (15–17). Many patients with diabetes have high levels of leptin (18–20), which may stimulate TSH synthesis by influencing the hypothalamic-pituitary-thyroid (HPT) axis through (JAK)-2/signal transduction and transcriptional activation (STAT)3 (15). In addition, hyperinsulinemia is common in people with T2DM, and insulin also affects the release of TRH and TSH (21, 22).

One report suggested that the risk of T2DM is positively associated with TSH levels and negatively associated with T3 and FT4 levels (23). A normal reduction in FT4 levels is associated with hyperglycemia and insulin resistance (24). However, other studies have shown that the FT4 level is positively correlated with the incidence of T2DM (25) and fasting blood glucose levels (26). Laclaustra and colleagues was the first to use the TFQI index to assess diabetes and diabetes-related mortality (7). The findings suggested that TSH or FT4 levels alone cannot explain the association between thyroid dysfunction and abnormal blood glucose. In our study, the central sensitivity index of thyroid hormone was used to evaluate the relationship between blood glucose and thyroid hormone. The results indicated that the decrease in thyroid hormone sensitivity was related to the increase in FPG, OGTT2hPG and HbA1c values and the decrease in HDL-C, FINS, OGTT2hINS and HOMA-β values.

In this study, insulin resistance was higher in the T2DM-SCH+ group than in the T2DM-SCH- group. There was no significant difference between the T2DM group and the T2DM-SCH+ group, in addition the T2DM-SCH+ group was accompanied by hyperinsulinemia. Similar to previous studies, the insulin resistance index was increased in patients with subclinical hypothyroidism compared with those with normal thyroid function (8, 27). Linear analysis of normal thyroid function showed that TSH was positively correlated with insulin resistance (28, 29). Velija-Asimi et al. (30) found that fasting insulin levels were significantly higher in the subclinical hypothyroid group than in the normal thyroid group, suggesting that fasting hyperinsulinemia may be an early manifestation of glucose metabolism disorder. A similar meta-analysis suggested a J-shaped relationship between T2DM and TSH. In the normal population, the risk of T2DM increased by 11% for every 1 mIU/L increase in TSH (3). The relationship between subclinical hypothyroidism and insulin resistance was demonstrated. However, the causal relationship between the two has not been fully proven, and the “common root” may be used as the reason for the explanation. Leptin is an important endogenous fat cell-derived protein that is involved in controlling food intake through its effect on the hypothalamus, leading to appetite suppression. Obesity is characterized by hyperleptinemia due to the development of leptin resistance (20). Obesity and elevated leptin levels are associated with insulin resistance and type 2 diabetes (31). As mentioned earlier, leptin can directly or indirectly regulate TRH, and leptin can also stimulate TSH synthesis. Additionally, TSH can also bind to the TSH receptor of preadipocytes, induce preadipocyte differentiation and adipocyte formation, promote obesity and lead to insulin resistance (32). Studies have shown that the level of hypersensitive C-reactive protein in subclinical hypothyroidism was significantly higher than that in the normal thyroid function group (33, 34), and hypersensitive C-reactive protein was significantly decreased after treatment with L-thyroxine (30). Subclinical hypothyroidism may lead to an increase in inflammatory cytokine release. This mediates the occurrence of inflammatory reactions, thus causing hyperinsulinemia and insulin resistance. The results of this study indicated that the BMI, waist circumference, waist-to-hip ratio and insulin resistance of the T2DM-SCH+ group were the same as those of the T2DM group, suggesting that elevation of TSH has a similar effect on insulin resistance in the presence or absence of T2DM. The increase in TSH levels may be caused by the adipocyte, leptin, inflammatory response and other pathways (15, 32, 33). In addition, in the T2DM-SCH+ group, due to the existence of insulin resistance, insulin compensatory functions increase, and failure to compensate will lead to an increased risk of diabetes.

In this study, the insulin resistance of the subclinical hypothyroidism group and normal thyroid function group was compared in the T2DM population, and the results indicated that there was no significant difference in insulin resistance between the two groups. Most studies have explored the effect of simple subclinical hypothyroidism on insulin resistance, while there are fewer studies on insulin resistance in patients with T2DM and subclinical hypothyroidism. Kouidhi et al. have shown that TSH is associated with insulin resistance in people with obesity and/or T2DM. In the diabetic nonobese group, there was no correlation between TSH and any other study parameters (35). In this study, the BMI of the T2DM population was dominated by overweight rather than obesity, so comparing insulin resistance in the subclinical hypothyroid group with the normal thyroid function group in the diabetic population did not suggest a clear difference. In another study of patients with type 1 diabetes, whether subclinical hypothyroidism is combined with type 1 diabetes had no significant effect on the difference in HbA1c and total insulin requirement in patients with diabetes (36). T2DM is a multi-factorial metabolic disorder with more grave consequences as compared to subclinical hypothyroidism. Results obtained in the T2DM subjects of the current study were similar to that obtained by Kouidhi et al. in T1DM. Thus, in this study, the insulin resistance of the population with diabetes in the subclinical hypothyroidism group was the same as that in the normal thyroid function group, which may be due to the coexistence of T2DM. Insulin resistance was masked by the metabolic disorder of diabetes, and subclinical hypothyroidism had no effect on insulin resistance. However, the effect of subclinical hypothyroidism on insulin resistance was fully shown in the normal blood glucose population.

## 5 Conclusion

In summary, our study demonstrates that subclinical hypothyroidism increases insulin resistance in people with normal blood glucose levels and that decreased thyroid hormone sensitivity is associated with the risk of developing diabetes. These results may contribute to further understanding of the interaction between thyroid hormone and glucose metabolism, more helpful understanding of the pathogenesis and treatment of thyroid disease and diabetes.This study also has certain limitations. We did not measure other measures such as leptin, and assessing leptin levels would be more helpful in understanding the relationship between SCH and insulin resistance.

## Data availability statement

The original contributions presented in the study are included in the article/supplementary material. Further inquiries can be directed to the corresponding author.

## Author contributions

WY performed the experiment and draft the manuscript. CJ, HW, YL and JL participated in the data collection. ZS designed the study in the revised manuscript. All authors contributed to the article and approved the submitted version.
